# The Italian Registry for Primary Immunodeficiencies (Italian Primary Immunodeficiency Network; IPINet): Twenty Years of Experience (1999–2019)

**DOI:** 10.1007/s10875-020-00844-0

**Published:** 2020-08-15

**Authors:** Vassilios Lougaris, Andrea Pession, Manuela Baronio, Annarosa Soresina, Roberto Rondelli, Luisa Gazzurelli, Alessio Benvenuto, Silvana Martino, Marco Gattorno, Andrea Biondi, Marco Zecca, Maddalena Marinoni, Giovanna Fabio, Alessandro Aiuti, Gianluigi Marseglia, Maria Caterina Putti, Carlo Agostini, Claudio Lunardi, Alberto Tommasini, Patrizia Bertolini, Eleonora Gambineri, Rita Consolini, Andrea Matucci, Chiara Azzari, Maria Giovanna Danieli, Roberto Paganelli, Marzia Duse, Caterina Cancrini, Viviana Moschese, Luciana Chessa, Giuseppe Spadaro, Adele Civino, Angelo Vacca, Fabio Cardinale, Baldassare Martire, Luigi Carpino, Antonino Trizzino, Giovanna Russo, Fausto Cossu, Raffaele Badolato, Maria Cristina Pietrogrande, Isabella Quinti, Paolo Rossi, Alberto Ugazio, Claudio Pignata, Alessandro Plebani

**Affiliations:** 1 Pediatrics Clinic and Institute for Molecular Medicine A. Nocivelli, Department of Clinical and Experimental Sciences, University of Brescia and ASST-Spedali Civili di Brescia, Piazzale Spedali Civili 1, 25123 Brescia, Italy; 2 Unit of Pediatrics, University of Bologna, St. Orsola University Hospital, Bologna, Italy; 3 Pediatrics Clinic, ASST- Spedali Civili of Brescia, Brescia, Italy; 4 Division of Pediatric Immunology and Rheumatology, Department of Public Health and Pediatrics, “Regina Margherita” Children Hospital, University of Turin, Turin, Italy; 5 Centro Malattie Autoinfiammatorie e Immunodeficienze- IRCCS Giannina Gaslini, Via Gaslini 5, 16147 Genoa, Italy; 6 Clinica Pediatrica, MBBM Foundation, University of Milano-Bicocca, Monza, Italy; 7 Department of Pediatric Hematology/Oncology, Fondazione IRCCS Policlinico San Matteo, Pavia, Italy; 8 Paediatric Department, ASST-Sette Laghi, “F. Del Ponte” Hospital, Varese, Italy; 9 Department of Internal Medicine, Fondazione IRCCS Ca’ Granda Ospedale Maggiore Policlinico, Milan, Italy; 10 San Raffaele Telethon Institute for Gene Therapy (SR-Tiget), IRCCS San Raffaele Scientific Institute, Milan, Italy; 11 Department of Paediatric Immunohematology, IRCCS San Raffaele Scientific Institute, Milan, Italy; 12 Vita Salute San Raffaele University, Milan, Italy; 13 Department of Pediatrics, Foundation IRCCS Policlinico San Matteo, University of Pavia, Pavia, Italy; 14 Department of Women’s and Children’s Health, Pediatric Hematology-Oncology Unit, University of Padova, Padova, Italy; 15 Center for Immunologic, Rheumatologic and Respiratory Diseases, Ca’ Foncello Hospital, Treviso, Italy; 16 Department of Medicine, University of Verona, Piazzale L.A. Scuro 10, 37134 Verona, Italy; 17 Department of Paediatrics, Institute for Maternal and Child Health-IRCCS “Burlo Garofolo”, Trieste, Italy; 18 Department of Medical, Surgical and Health Sciences, University of Trieste, Trieste, Italy; 19 Paediatric Hematology Oncology Unit, Azienda Ospedaliero-Universitaria di Parma, Parma, Italy; 20 Department of Experimental and Clinical Medicine, University of Florence, Florence, Italy; 21 Section of Pediatrics Immunology and Rheumatology, Department of Pediatrics, University of Pisa, Pisa, Italy; 22 Immunoallergology Unit, AOU Careggi, University of Florence, Florence, Italy; 23 Department of Pediatric Immunology, Jeffrey Modell Center for Primary Immunodeficiency, Anna Meyer’s Hospital, University of Florence, Florence, Italy; 24 Clinica Medica, Dipartimento di Scienze Cliniche e Molecolari, Università Politecnica delle Marche e Azienda Ospedali Riuniti, Ancona, Italy; 25 Section of Traslational Medicine, Department of Medicine and Sciences of Aging, G. d’Annunzio University, Chieti, Italy; 26 Pediatrics Department, Umberto I Hospital, Rome, Italy; 27 Sapienza University, Rome, Italy; 28 Unit of Immunology and Infectious Diseases, Academic Department of Pediatrics, Bambino Gesù Children’s Hospital, Rome, Italy; 29 Department of Pediatrics, Policlinico Tor Vergata, Tor Vergata University, Rome, Italy; 30 Department of Clinical and Molecular Medicine, Sapienza University, Rome, Italy; 31 Department of Translational Medical Sciences, Allergy and Clinical Immunology Center for Basic and Clinical Immunology Research (CISI), University of Naples Federico II, Naples, Italy; 32 Ospedale Vito Fazzi, Lecce, Italy; 33 Department of Biomedical Sciences and Human Oncology, Section of Internal Medicine and Clinical Oncology, University of Bari Medical School, Bari, Italy; 34 Department of Pediatrics and Emergency, Pediatric Allergy and Pulmunology Unit, Azienda Ospedaliera-Universitaria “Consorziale-Policlinico”, Ospedale Pediatrico Giovanni XXIII, Bari, Italy; 35 Pediatric Unit, “Monsignor Dimiccoli” Hospital, Barletta, Italy; 36 Pediatrics Unit, “Annunziata” Hospital, Cosenza, Italy; 37 Department of Pediatric Hematology and Oncology, ARNAS Civico Di Cristina and Benfratelli Hospital, Palermo, Italy; 38 Haematology/Oncology Unit, Department of Clinical and Experimental Medicine, University of Catania, Catania, Italy; 39 2nd Pediatric Clinic, Antonio Cao Hospital, University of Cagliari, Cagliari, Italy; 40 Department of Pediatrics, Fondazione IRCCS Ca’ Granda Ospedale Maggiore Policlinico, University of Milan, Milan, Italy; 41 Department of Molecular Medicine, Sapienza University, Rome, Italy; 42 Institute of Child and Adolescent Health, Bambino Gesù Children’s Hospital, IRCCS, Rome, Italy; 43 Pediatric Section, Department of Translational Medical Science, Federico II University, Naples, Italy

**Keywords:** Primary immunodeficiencies, patient registry

## Abstract

Primary immunodeficiencies (PIDs) are heterogeneous disorders, characterized by variable clinical and immunological features. National PID registries offer useful insights on the epidemiology, diagnosis, and natural history of these disorders. In 1999, the Italian network for primary immunodeficiencies (IPINet) was established. We report on data collected from the IPINet registry after 20 years of activity. A total of 3352 pediatric and adult patients affected with PIDs are registered in the database. In Italy, a regional distribution trend of PID diagnosis was observed. Based on the updated IUIS classification of 2019, PID distribution in Italy showed that predominantly antibody deficiencies account for the majority of cases (63%), followed by combined immunodeficiencies with associated or syndromic features (22.5%). The overall age at diagnosis was younger for male patients. The minimal prevalence of PIDs in Italy resulted in 5.1 per 100.000 habitants. Mortality was similar to other European registries (4.2%). Immunoglobulin replacement treatment was prescribed to less than one third of the patient cohort. Collectively, this is the first comprehensive description of the PID epidemiology in Italy.

## Introduction

Primary immunodeficiencies comprise a heterogeneous group of rare disorders, characterized by a variety of possible immunological alterations that influence the age at onset of disease for affected individuals [1, 2]. Considering the low number of affected patients, awareness among clinicians and healthcare professionals may frequently not be optimal. In addition, misdiagnosis or delay in diagnosis may negatively influence both prognosis and outcome for affected patients.

Organized registries represent an efficient and well-defined instrument for better characterization and understanding of rare diseases, with positive impact on the clinical management of affected patients and on the comprehension of the natural history of the disease [3, 4]. In the last few decades, registries have been created for primary immunodeficiencies in several countries around the world in order to better define the distribution and features of patients affected with these disorders [5–19].

In 1999, the Italian primary immunodeficiency network (IPINet) was established within AIEOP (Italian Association of Pediatric Haematology and Oncology), based on a nationwide effort aimed at better defining the distribution and features of patients with PIDs in Italy. After 20 years of operative experience, a progressive increase in participating centers led to the current status of 60 PID centers and 3352 registered patients. In this paper, we describe the IPINet experience in the field of PIDs in Italy and offer a comprehensive description of PID distribution, as well as PID’s minimal prevalence in Italy.

## Materials and Methods

Patients’ data were collected from the online database of the IPINet (Italian primary immunodeficiency network) registry (https://www.aieop.org/web/) through the CINECA platform according to the AIEOP model previously described [20]. Medical centers following pediatric and adult PID patients, as well as members of the IPINet network, recorded data for patients followed at their clinic since diagnosis based on the ESID diagnostic criteria with annual update during follow-up. The IPINet web-site contains freely available diagnostic and therapeutic guidelines for the different forms of PIDs. The IPINet registry and related inform consent forms have been approved from the local ethical committee. A questionnaire including relevant clinical and immunological features was compiled annually upon enrollment. The dataset included blood exams (differential blood count, routine biochemistry, immunoglobulin serum levels, lymphocyte subsets), imaging data (chest X-rays, lung and sinus CT scans, abdomen ultrasonography etc.), treatment details (Ig replacement treatment, antibiotic prophylaxis etc.), infectious history data (type of infection, type of pathogen, if isolated, type of treatment, duration of infection, admission or not), cancer data (type of cancer, treatment), and outcome (alive, dead). Every referral center has access to the data regarding his/her center. For each sub-registry (for example XLA, AAR, CVID etc.), there is a coordinator that has access to all relative data for this subgroup of PID. Finally, the central operative office (CINECA) can extrapolate all necessary data based on specific request from the IPINet members, once the request has been approved by the IPINet steering group. Genetic testing results were not originally included in this dataset. This collective effort was initiated in 1999. Data included in this study were collected for the period 1999–2019.

## Results

### Distribution of PIDs in the IPINet Registry

**Fig. 1:**
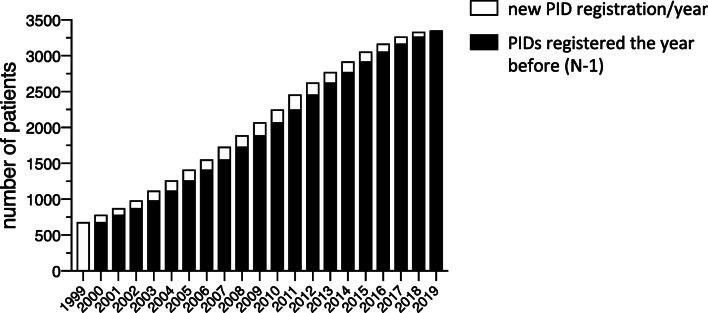
PID registration in the IPINet registry. Annual numbers for new (white bars) and patients registered the year before (N-1) (black bars) for the period 1999–2019

**Fig. 2:**
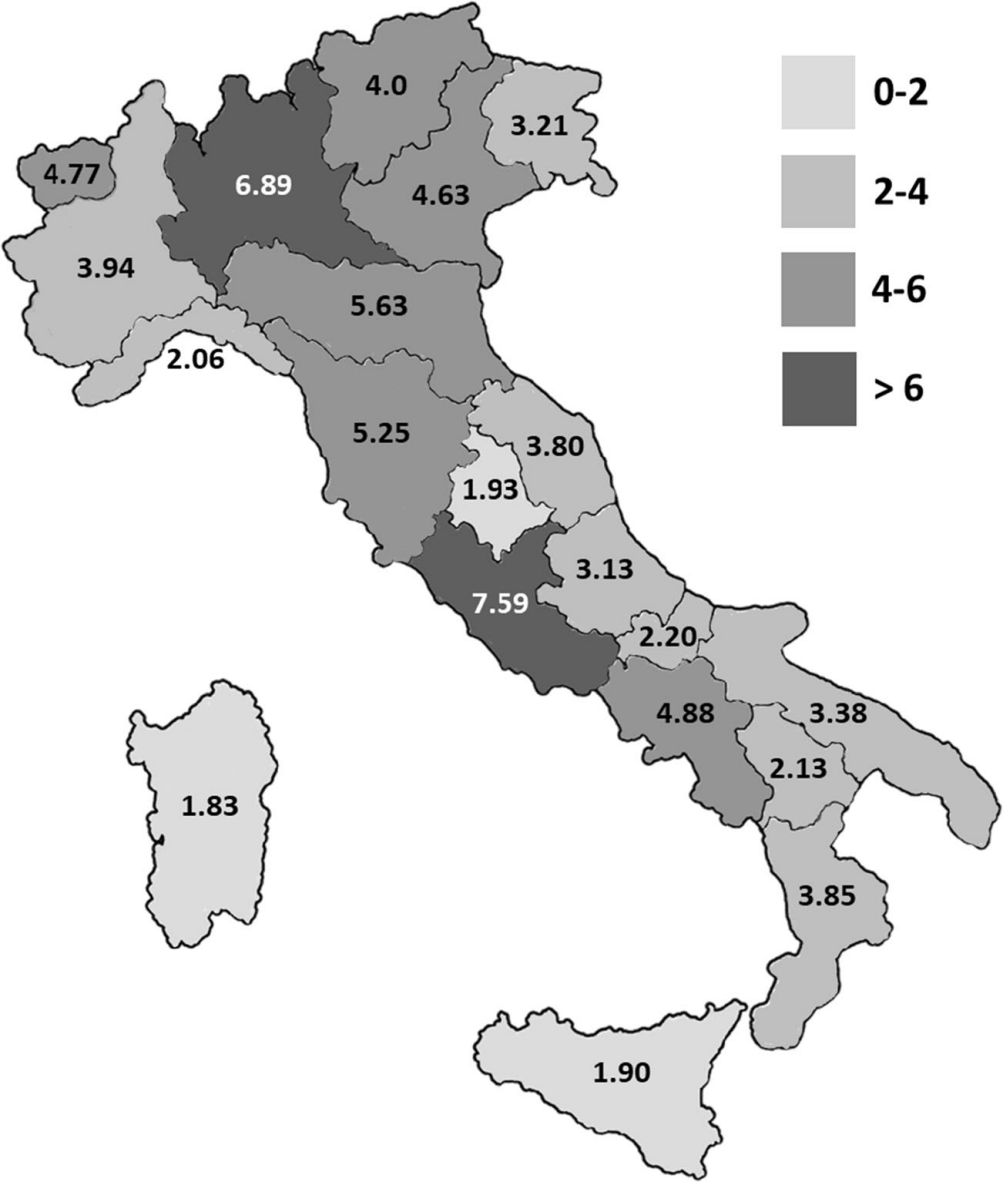
Regional minimal prevalence of PIDs in Italy. Geographic distribution of PIDs in the Italian territory based on patients’ residence (data refer to 2019). Darker shades of gray areas correspond to higher minimal prevalence of PIDs. Data are expressed as minimal prevalence calculated per 10^5^ inhabitants

**Fig. 3:**
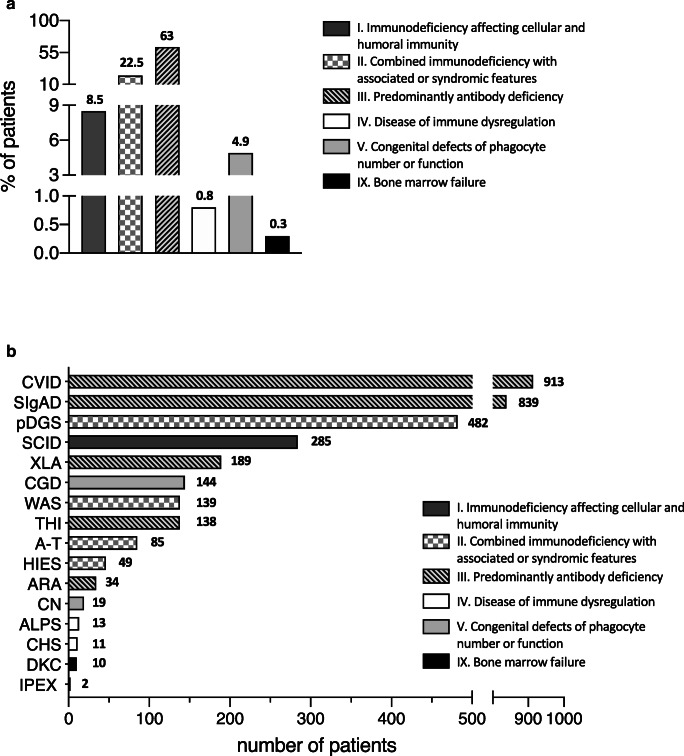
PID distribution and characteristics in the IPINet registry. **a** Overall PID distribution in Italy (percentages) based on the latest IUIS classification (2019). **b** Detailed PIDs’ distribution in the IPINet registry (number of patients). Patterns identify six groups based on the latest IUIS classification

The IPINet registry currently includes 3352 patients affected with PIDs (male vs female: 59.6% vs 40.4%). A progressive increase in the number of registered patients was noted over these 20 years, as shown in Fig. 1. Evaluation of the regional distribution pattern in Italy revealed that the minimal prevalence of PIDs based on patients’ residence is higher in Lombardy and Lazio, followed by Emilia Romagna, Tuscany, and Campania (Fig. 2). The distribution of PIDs, based on the latest IUIS classification [1, 2] within the IPINet registry, is depicted in Fig. 3a. Almost two-thirds of registered patients are affected with predominantly antibody deficiencies (63%), in line with previously published data [5–18]. Combined immunodeficiencies with associated or syndromic features represent a smaller portion of the IPINet registry (22.5%), followed by immunodeficiencies affecting cellular and humoral immunity (8.5%) and congenital defects of phagocyte number or function (4.9%) (Fig. 3a).

More in detail (Fig. 3b), predominantly antibody deficiencies within the IPINet registry can be divided in Common Variable Immunodeficiency (CVID) (27.2%, 913 patients), Selective IgA Deficiency (SIgAD) (25%, 839 patients), X-linked Agammaglobulinemia (XLA) (5.6%, 189 patients), Transient Hypogammaglobulinemia of the Infancy (THI) (4.1%, 138 patients), and Autosomal Recessive Agammaglobulinemia (ARA) (1%, 34 patients) (Fig. 3b and Table 1). Combined immunodeficiencies with associated or syndromic features included patients affected with Wiskott-Aldrich Syndrome (WAS) (4.2%, 139 patients), partial DiGeorge Syndrome (pDGS) (14.4%, 482 patients), Ataxia-Telangiectasia (A-T) (2.5%, 85 patients), and Hyper-IgE Syndrome (HIES) (1.5%, 49 patients) (Fig. 3b and Table 1). The group of immunodeficiency affecting cellular and humoral immunity included only SCID/CID patients (8.5%, 285 patients). Congenital defects of phagocyte number or function included Chronic Granulomatous Disease (CGD) (4.3%, 144 patients) and Congenital Neutropenia (CN) (0.6%, 19 patients). We found a small amount of patients affected with diseases of immune dysregulation that comprised Chediak-Higashi Syndrome (CHS) (0.3%, 11 patients), Autoimmune Lymphoproliferative Syndrome (ALPS) (0.4%, 13 patients), and Immunodysregulation Polyendocrinopathy Enteropathy X-linked (IPEX) (0.1%, 2 patients) (Fig. 3b and Table 1). Finally, in the group of bone marrow failure, a small number of patients with Dyskeratosis Congenita (DKC) was registered (0.3%, 10 patients) (Fig. 3b and Table 1).

**Table 1:** PID distribution in the IPINet registry. Detailed distribution of affected patients in the IPINet registry based on disease, sex, and age at diagnosis classified according to the latest IUIS classification (2019) [1, 2]

PID subgroups	PID diagnosis		Number of patients (%)	Median age at diagnosis (years) (range)	Number of pts < 18 years (%)	Number of pts > 18 years (%)	Alive pts at last follow-up (%)	Dead pts (%)	Pts lost during follow-up (%)	Pts diagnosed before 1999 (%)	Median age at diagnosis for pts diagnodes before 1999 (years) (range)	Pts diagnosed from 1999 to 2019 (%)	Median age at diagnosis for pts diagnodes from 1999 to 2019 (years) (range)
1	Severe Combined Immunodeficiency (SCID)		285 (8.5)	0.4 (0–22)	284 (99.6)	1 (0.4)	240 (84.2)	39 (13.7)	6 (2.1)	39 (13.7)	0.4 (0–11)	246 (86.3)	0.4 (0–22)
		Male	166 (58.2)	0.4	(0–11)	166 (58.5)	0137 (57.1)	26 (66.7)					
		Female	119 (41.8)	0.4 (0–22)	118 (41.5)	1 (100)	103 (42.9)	13 (33.3)					
II	Wiskott-Aldrich Syndrome (WAS)		139 (4.2)	0.9 (0–54)	135 (97.1)	4 (2.9)	119 (85.6)	14 (10.1)	6 (4.3)	34 (24.5)	0.8 (0–9)	105 (75.5)	0.9 (0–54)
		Male	138 (99.3)	0.9 (0–54)	134 (99.3)	4 (100)	118 (99.2)	14 (100)					
		Female	1 (0.7)	0.1 (0.1–0.1)	1 (0.7)	0	1 (0.8)	0					
	partial DiGeorge Syndrome (pDGS)		482 (14.4)	0.7 (0–46)	463 (96.1)	19 (3.9)	466 (96.7)	15 (3.1)	1 (0.2)	31 (6.4)	1.5 (0–18)	451 (93.6)	0.6 (0–46)
		Male	263 (54.6)	0.4 (0–46)	258 (55.7)	5 (26.3)	252 (54.1)	10 (66.7)					
		Female	219 (45.4)	0.7 (0–42)	205 (44.3)	14 (73.7)	214 (45.9)	5 (33.3)					
	Ataxia-Telangiectasia (A-T)		85 (2.5)	5.7 (0–34)	79 (92.9)	6 (7.1)	76 (89.4)	8 (9.4)	1 (1.2)	13 (15.3)	4.9 (0–24)	72 (84.7)	5.7 (1–34)
		Male	38 (44.7)	5.2 (2–34)	35 (44.3)	3 (50)	33 (43.4)	4 (50)					
		Female	47 (55.3)	5.9 (0–28)	44 (55.7)	3 (50)	43 (56.6)	4 (50)					
	Hyper-IgE Syndrome (HIES)		49 (1.5)	11 (0–46)	35 (71.4)	14 (28.6)	44 (89.8)	4 (8.2)	1 (2)	6 (12.2)	10.5 (0–12)	43 (87.8)	12.8 (0–46)
		Male	27 (55.1)	12.8 (0–46)	17 (48.6)	10 (71.4)	24 (54.5)	2 (50)					
		Female	22 (44.9)	11 (0–37)	18 (51.4)	4 (28.6)	20 (45.5)	2 (50)					
III	Common Variable Immunodeficiency (CVID)		913 (27.2)	30.3 (0–83)	277 (30.3)	636 (69.7)	872 (95.5)	29 (3.2)	12 (1.3)	221 (24.2)	25.4 (0–71)	692 (75.8)	33.4 (0–83)
		Male	451 (49.4)	24.9 (0–83)	174 (62.8)	277 (43.6)	430 (49.3)	16 (55.2)					
		Female	462 (50.6)	35.4 (0–83)	103 (37.2)	359 (56.4)	442 (50.7)	13 (44.8)					
	Selective IgA Deficiency (SIgAD)		839 (25)	5.6 (0–70)	804 (95.8)	35 (4.2)	769 (91.7)	1 (0.1)	69 (8.2)	73 (8.7)	5.6 (0–19)	766 (91.3)	5.6(0–70)
		Male	452 (53.9)	5.5 (0–55)	439 (54.6)	13 (37.1)	412 (53.6)	0					
		Female	387 (46.1)	5.7 (0–70)	365 (45.4)	22 (62.9)	357 (46.4)	1 (100)					
	X-linked Agammaglobulinemia (XLA)		189 (5.6)	2.7 (0–52)	176 (93.1)	13 (6.9)	172 (91)	12 (6.3)	5 (2.7)	88 (46.6)	3.2 (0–40)	101 (53.4)	2.5 (0–52)
		Male	189 (100)	2.7 (0–52)	176 (100)	13 (100)	172 (100)	12 (100)					
		Female	0	/	0	0	0	0					
	Transient Hypogammaglobulinemia of Infancy (THI)		138 (4.1)	1 (1–5)	138 (100)	0	134 (97.1)	0	4 (2.9)	5 (3.6)	1.3 (0–3)	133 (96.4)	1 (0–5)
		Male	91 (65.9)	1 (1–5)	91 (65.9)	0	89 (66.4)	0					
		Female	47 (34.1)	1.1 (1–3)	47 (34.1)	0	45 (33.6)	0					
	Autosomal Recessive Agammaglobulinemia (ARA)		34 (1)	2 (0–58)	26 (76.5)	8 (23.5)	32 (94.1)	2 (5.9)	0	17 (50)	5.1 (0–58)	17 (50)	1.3 (0–44)
		Male	20 (58.9)	1.6 (0–58)	15 (57.7)	5 (62.5)	18 (56.2)	2 (100)					
		Female	14 (41.2)	2.5 (0–45)	11 (42.3)	3 (37.5)	14 (43.8)	0					
IV	Chediak-Higashi Syndrome (CHS)		11 (0.3)	1.7 (0–11)	11 (100)	0	9 (81.8)	1 (9.1)	1 (9.1)	2 (18.2)	3.9	9 (81.8)	1.7 (0–11)
		Male	4 (36.4)	0.9 (0–3)	4 (36.4)	0 3 (33.3)	1 (100)						
		Female	7 (63.6)	2 (1–11)	7 (63.6)	0	6 (66.7)	0					
	Immunodysregulation Polyendocrinopathy Enteropathy X-linked (IPEX)		2 (0.1)	0.4 (0–9)	2 (100)	0 1 (50)		1 (50)	0	0	/	2 (100)	0.3 (0–9)
		Male	2 (100)	0.4 (0–9)	2 (100)	0	1 (100)	1 (100)					
		Female	0	/	0	0	0	0					
	Autoimmune Lymphoproliferative Syndrome(ALPS)		13 (0.4)	8.2 (1–18)	12 (92.3)	1 (7.7)	13 (100)	0	0	0	/	13 (100)	7.7 (1–18)
		Male	6 (46.2)	6.8 (2–18)	5 (41.7)	1 (100)	6 (46.2)	0					
		Female	7 (53.8)	7.7 (1–13)	7 (58.3)	0	7 (53.8)	0					
V	Congenital Neutropenia (CN)		19 (0.6)	0.3 (0–14)	19 (100)	0	16 (84.2)	2 (10.5)	1 (5.3)	0	/	19 (100)	0.3 (0–14)
		Male	10 (52.6)	0.8 (0–14)	10 (52.6)	0	9 (56.2)	1 (50)					
		Female	9 (47.4)	0.2 (0–2)	9 (47.4)	0	7 (43.8)	1 (50)					
	Chronic Granulomatous Disease (CGD)		144 (4.3)	2.2 (0–48)	139 (96.5)	5 (3.5)	130 (90.3)	12 (8.3)	2 (1.4)	50 (34.7)	2.4 (0–23)	94 (65.3)	2.2 (0–48)
		Male	136 (94.4)	2.2 (0–48)	131 (94.2)	5 (100)	122 (93.8)	12 (100)					
		Female	8 (5.6)	4.7 (2–17)	8 (5.8)	0	8 (6.2)	0					
IX	Dyskeratosis congenita (DKC)		10 (0.3)	7 (2–11)	10 (100)	0	10 (100)	0	0	0	/	10 (100)	6.7 (2–11)
		Male	6 (60)	7.2 (2–11)	6 (60)	0	6 (60)	0					
		Female	4 (40)	6.5 (3–8)	4 (40)	0	4 (40)	0					
	Total		3352 (100)	5 (0–83)	2610 (77.9)	742 (22.1)	3103 (92.6)	140 (4.2)	109 (3.2)	579 (17.3)	6 (0–71)	2773 (82.7)	4 (0–83)
		Male	1999 (59.6)	4 (0–82)	1663 (63.7)	336 (45.3)	1832 (59)	101 (72.1)					
		Female	1353 (40.4)	6 (0–83)	947 (36.3)	406 (54.7)	1271 (41)	39 (27.9)					

I—Immunodeficiency affecting cellular and humoral immunity

II—Combined immunodeficiency with associated or syndromic features

III—Predominantly antibody deficiency

IV—Disease of immune dysregulation

V—Congenital defects of phagocyte number or function

IX—Bone marrow failure

### Age at Diagnosis of PID Patients in the IPINet Registry

Age at diagnosis was younger among male patients compared with the female ones (4 vs 6 years respectively; median values) (Table 1). More in detail, among predominantly antibody deficiencies, CVID is the only one with a diagnosis more frequent in adult age (> 18 years) (636 patients) rather than in childhood (< 18 years) (277 patients) (Table 1), with a median age at diagnosis of 25 years (Table 1). XLA on the other hand was mainly diagnosed before adulthood (176 out of 189 patients) (median age at diagnosis: 3 years), and a similar trend was observed for both ARA (26 out of 34) (median age at diagnosis: 2 years) and SIgAD (804 out of 839 patients) (median age at diagnosis: 5 years) (Table 1). By definition, THI was only diagnosed during childhood, with a median age at diagnosis of 1 year. SCID/CID were almost exclusively diagnosed in childhood (284 out of 285 patients), with a median age at diagnosis of 0 year (Table 1). Combined immunodeficiency with associated or syndromic features (WAS, pDGS, A-T) followed the same pattern of diagnosis before adulthood (Table 1). In slight contrast with this trend, HIES was diagnosed during adulthood in almost one-third of affected patients (14 out of 49 patients) (median age at diagnosis: 11 years) (Table 1). Finally, diseases of immune dysregulation, congenital defects of phagocyte number or function, and bone marrow failure were all diagnosed in childhood (Table 1). Of note, median age of diagnosis before and after the establishment of the IPINet registry did not show significant differences in most cases (Table 1).

### Treatment and Survival of PID Patients in the IPINet Registry

Less than one-third of the patient cohort was under regular immunoglobulin replacement treatment, with an evident prevalence of the endovenous route (20.6%) contrary to what observed in other countries, where the subcutaneous route is preferred [5–19].

**Fig. 4:**
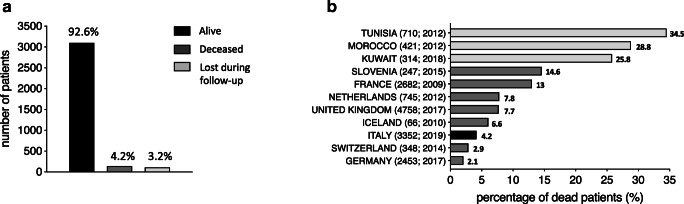
Death rate of PID patients in the IPINet registry. **a** Number and percentages of alive, deceased, and lost to follow-up patients during the 20-year follow-up period (patients lost to follow-up patients without updated data in the registry over the years). **b** Comparison of IPINet PID death percentages with previously published PID registries of other countries (Italy: black; Europe: gray; other countries: light gray). Parentheses next to each country name correspond to number of patients followed by year of analysis (number of patients; year of analysis)

The majority of patients within the IPINet registry (92.6%) were alive during the last follow-up with 4.2% of deaths (140 patients) (Fig. 4a). With the exception of pDGS where the majority of deceased patients were males (66.7%), no significant sex-related differences were observed for the other PIDs (Table 1). Comparison with available data from other PID registries showed that this percentage of mortality is in line with several European registries, remaining inferior to the high mortality rates reported from Tunisia, Morocco, Kuwait, Slovenia, and France (Fig. 4b). Finally, 3.2% of patients were lost during follow-up (Fig. 4a).

### Prevalence of PIDs in Italy

The real prevalence of PIDs is not well defined and may depend upon various factors, such as form of PID, ethnic background, and consanguinity [5–18]. In recent years for example, the introduction of newborn screening (NBS) for SCID/CID in several countries has redefined the prevalence of these disorders [21].

**Fig. 5:**
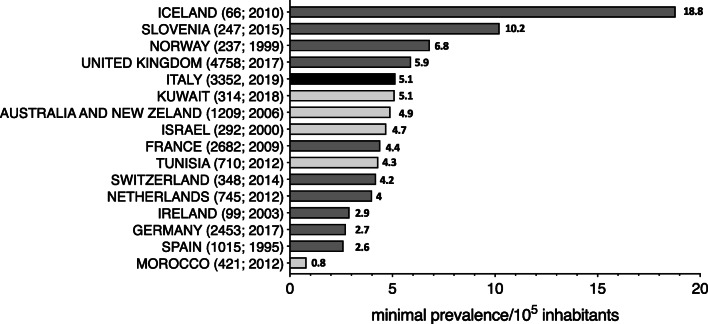
Minimal prevalence of PIDs in Italy and comparison with other countries. Comparison of minimal prevalence of PIDs in Italy calculated on alive patients with previously published data from other PID registries (Italy: black; Europe: gray; other countries: light gray). Parentheses next to each country name correspond to number of patients followed by year of analysis (number of patients; year of analysis)

Considering the large cohort of the IPINet registry, we wanted to calculate the minimal prevalence of PIDs in the Italian territory. The minimal prevalence of PIDs in Italy in 2019 is higher (5.1 per 100.000) than the one reported from most European PID registries, with the exception of published data from Iceland, Slovenia, Norway, and the United Kingdom (Fig. 5). It also resulted higher than the PID minimal prevalence reported from Morocco, Spain, Germany, Ireland, Netherlands, Switzerland, Tunisia, and France while it was similar than the ones reported from Kuwait, Australia and New Zealand, and Israel (Fig. 5).

The minimal prevalence of predominantly antibody deficiencies within the IPINet registry (3.28 per 100.000) was higher from what reported so far from most registries, with the exception of the United Kingdom and Iceland (Table 2). The minimal prevalence of combined immunodeficiencies with associated or syndromic features within the IPINet registry (1.17 per 100.000) resulted again higher when compared with the minimal prevalence from other registries, with the exception of Kuwait and Iceland (Table 2). Regarding the remaining PIDs, their minimal prevalence in Italy showed an intermediate collocation in relation to other countries (Table 2).

**Table 2:** Minimal prevalence of PIDs in Italy and comparison with other countries. Minimal prevalence (per 10^5^ inhabitants) of PIDs in Italy calculated on alive patients based on the latest IUIS classification (2019) and comparison with previously published data from other national PID registries. Numbers in parentheses represent total number of registered patients (bold) and total number of alive patients (italic)

		Italy (**3352**; *3103***)**	France [10] (**3083**; *2682***)**	Morocco[11] (**421**; *304***)**	Iceland [13] **(66**; *62***)**	Switzerland [14] (**348**; *338*)	Tunisia [15] **(710**; *465***)**	The United Kingdom [17] **(4758**; *4297***)**	Kuwait [19] (**314**; *233***)**
I	Immunodeficiency affecting cellular and humoral immunity	0.40	0.21	0.12	/	/	0.82	0.50	1.23
II	Combined immunodeficiency with associated or syndromic features	1.17	0.51	0.26	2.80	0.35	0.26	0.49	1.25
III	Predominantly antibody deficiency	3.28	2.09	0.22	7.50	2.65	1.12	3.92	1.18
IV	Disease of immune dysregulation	0.04	/	0.01	/	0.10	0.19	0.14	0.61
V	Congenital defects of phagocyte number or function	0.24	0.84	0.19	1.90	0.36	1.13	0.27	0.33
VI	Defects of intrinsic and innate immunity	/	/	0.03	/	0.09	/	0.06	/
VII	Autoinflammatory disorders	/	/	0.03	/	0.15	/	0.04	0.02
VIII	Complement deficiency	/	0.02	0.04	5.70	0.19	0.02	0.85	0.48
IX	Bone marrow failure	0.02	/	/	/	/	/	/	/

## Discussion

The minimal prevalence of PIDs is still not well defined, possibly because it may be influenced by several factors such as ethnicity, consanguinity, age, and awareness among physicians. National registries have been shown to be of great help in better defining the minimal prevalence and natural history of rare diseases, PIDs included [5–19]. In this study, we report for the first time on the minimal prevalence and distribution of PIDs in Italy. IPINet, a national network on primary immunodeficiencies, was established in 1999 and now involves 60 medical centers following pediatric and adult patients throughout the Italian territory.

During the 20-year operative experience, IPINet has become an important PID registry with 3352 patients enrolled and with a continuous increasing trend of new cases/year. Upon the initial registration, physicians following PID patients update their clinical and immunological data at least once every year. A regional pattern of distribution was observed in this registry with the higher minimal prevalence of PID patients registered in two regions (Lombardy and Lazio). As reported for several other European PID registries, predominantly antibody deficiencies represent the most frequent forms of PIDs in the IPINet registry, followed by combined immunodeficiencies with associated or syndromic features and immunodeficiencies affecting cellular and humoral immunity. Two-thirds of registered patients are males and their median age at diagnosis was younger when compared with the female ones. Since IPINet comprises both pediatric and adult PID centers, almost one-fifth of registered patients were diagnosed in adulthood, most of which affected by predominantly antibody deficiencies.

This effort led to the calculation of PID minimal prevalence in Italy to be 5.1/100.000 habitants, which was higher when compared with most other PID registries, with the exception of Iceland and Slovenia [5–19]. The IPINet prevalence of predominantly antibody deficiencies is higher when compared with most of the other published PID registries. A similar trend was also observed for combined immunodeficiencies with associated or syndromic features.

Published data from national PID registries regarding survival of affected patients are limited. A small number of patients resulted deceased at the last follow-up (4.2%), in clear contrast with the data of registries from Tunisia, Morocco, and Kuwait [11, 15, 19]. This discrepancy may be largely due to the differences in minimal prevalence of several forms of PIDs in these countries, caused by the elevated consanguinity rate.

The data from our registry presented some limitations. First of all, in its original form, the IPINet database did not offer the possibility to include genetic data for registered patients, an aspect that will change in the revised version of the database. Secondly, disorders of the immune system such as autoinflammatory disorders and familial hemophagocytosis (FHL) are already included in distinct national registries, and thus, the overall numbers may be higher than the ones presented here. The same limitation refers to therapeutic options such as hematopoietic stem cell transplantation (HSCT) and gene therapy for which national registries are already operative.

## Conclusions

Since the establishment of IPINet, this is the first detailed description of PID prevalence and distribution in Italy over a 20-year period. The continuous annual increase of registered patients underlines a progressive improvement of PID awareness among physicians, although there is certainly a need for additional efforts in this direction. The IPINet registry appears to be one of the most numerous PID registries published to date, and continuous work and improved database organization in this registry will hopefully help to further ameliorate future diagnosis and management of PID patients in Italy.
